# Nurses’ attitudes towards the implementation of the Mother-Baby Friendly Initiative in selected primary healthcare facilities at Makhuduthamaga Municipality, Limpopo province

**DOI:** 10.4102/curationis.v42i1.1929

**Published:** 2019-03-05

**Authors:** Siyabulela E. Mgolozeli, Hilda N. Shilubane, Lunic B. Khoza

**Affiliations:** 1 Department of Advanced Nursing Science, University of Venda, Thohoyandou, South Africa

## Abstract

**Background:**

The implementation of the Mother-Baby Friendly Initiative (MBFI) strategy demonstrated its capabilities to improve global children’s health and maternal survival. However, its implementation in primary healthcare (PHC) facilities remains a challenge as many nurses are reluctant to adopt it for the improvement of child and maternal healthcare services in their respective clinics.

**Objectives:**

The primary objective of this study was to determine the attitudes of nurses towards the implementation of the MBFI in selected PHC facilities in the Makhuduthamaga Municipality, Limpopo province.

**Method:**

This study used a quantitative, descriptive design, and all respondents were conveniently sampled. A self-administered questionnaire was used to collect data. One-hundred and seventy-seven questionnaires were distributed, and 153 nurses responded and completed the questionnaire. The Statistical Package for Social Sciences version 23 was used to analyse data.

**Results:**

Results show that the majority of nurses (professional nurses [PNs] = 65, 78%; enrolled nurses [ENs] = 18, 72%; enrolled nursing auxiliaries [ENAs] = 23, 51%) had a positive attitude towards the MBFI strategy implementation as they agreed that it increased breastfeeding rates. Most PNs (*n* = 58, 70%) and ENs (*n* = 15, 60%) showed positive attitudes towards exclusive breastfeeding (EBF) as they agreed that it was the ideal feeding option for any child, and most ENAs (*n* = 38, 84%) showed a negative attitude as they disagreed that EBF was the ideal feeding option for any child.

**Conclusion:**

In this study, most PNs and ENs had a positive attitude in all the aspects that determined their attitudes towards MBFI strategy implementation. A concern is the fact that most ENAs showed negative attitudes in almost all the statements that were in line with the principles of MBFI, as they disagreed in most aspects. Therefore, this study recommends that on-going orientation and trainings should be offered to all nurses including ENAs to equip them with information that can assist in changing their attitudes towards MBFI implementation in PHC facilities.

## Introduction

Globally, suboptimal breastfeeding practices are estimated to be responsible for 800 000 child deaths annually (Piwoz & Huffman 2015:373). The United Nations International Children’s Emergency Fund (UNICEF) (2015:34) reports that globally, 38% of infants are exclusively breastfed for 6 months but only 31% in sub-Saharan Africa. The most common diseases that contribute to the high infant mortality rate in sub-Saharan Africa include stunting, vomiting and diarrhoea, and these could be prevented through breastfeeding. It has been documented that if breastfeeding uptake can be increased throughout sub-Saharan Africa, it can prevent approximately 40% of all under-five deaths (Azuine et al. 2015:13). In South Africa, breastfeeding rates remain relatively low (8%), with only 1.5% of infants being exclusively breastfed from birth to 6 months (Shisana et al. 2013:18). In response to low breastfeeding rates and increased child mortality rates worldwide, in 1991, the WHO launched the Baby-Friendly Hospital Initiative (BFHI) to promote and support breastfeeding by ensuring that all women breastfeed their babies and that they receive comprehensive support regarding breastfeeding (Department of Health [DoH] 2014:33; UNICEF/WHO 2018:2).

Initially, the BFHI focused on the 10 steps for successful breastfeeding, and the South African government renamed it the ‘Mother-Baby Friendly Initiative’ (MBFI) to include the mother-friendly component, the human immunodeficiency virus (HIV) and the code of marketing for breast milk substitutes (DoH 2014:33). The renaming of BFHI to MBFI intended to shift the initiative away from a hospital-based focus and expand it to all primary healthcare (PHC) facilities offering child and maternal health services. The renaming of BFHI to MBFI linked to Resolution 9 of the Tshwane Declaration (DoH 2014:33), which calls for community-based interventions to promote, protect and support breastfeeding (Du Plessis 2013:125). The implementation of this initiative in public and private health facilities has increased breastfeeding rates by more than 20% throughout the world (Munn et al. 2016:685; Zakarija Grković et al. 2017:e12249). The increase of breastfeeding rates is associated with an improved survival rate for infants and improved maternal outcomes such as the reduction of post-partum haemorrhage and ovarian cancers associated with the lack of breastfeeding (Nadeem et al. 2017:89). Also, breastfeeding is associated with many benefits for the mother including bonding with the infant, prevention of post-partum haemorrhage, child-distancing, calming the mother and being cost-effective (Dieterich et al. 2013:31).

Although exclusive breastfeeding (EBF) and complementary feeding are regarded as lifesaving solutions to our tragedy of infant mortality, the acceptance of EBF for the first 6 months of a baby’s life and the introduction of complementary feeding from 6 months is still a major challenge in our country. In 2011, the National Department of Health (NDoH) called for the preparation of all health facilities for MBFI accreditation as a way to accelerate the achievement of Millennium Development Goals (MDGs) 1, 4 and 5 (DoH 2014:33). Unfortunately, South Africa did not attain these MDG goals, and the implementation of MBFI was considered a relevant strategy to help the country to achieve Sustainable Development Goals 1, 2 and 3, as it has proven its capabilities in improving child and maternal health. While the MBFI strategy is reported as the best strategy for improvement of child and maternal health, the implementation of this strategy in PHC facilities remains a challenge, as most nurses are reluctant to adopt the WHO/UNICEF global steps for successful breastfeeding for the improvement of child and maternal healthcare services.

The researcher is a trained MBFI assessor who was tasked with conducting MBFI appraisal reports for Makhuduthamaga Municipality PHC facilities in 2015. The facility appraisals were conducted to prepare the facilities for MBFI national assessments. Many facilities did not perform well during the appraisal reports as many nurses showed little or no interest at all towards MBFI appraisals. In some facilities nurses reported that they were against the MBFI implementation as they felt it added more burden to their workload, and some demonstrated negative attitudes towards promotion, support and protection of breastfeeding. In antenatal clinics there were no structured breastfeeding teaching aids, and breastfeeding counselling was not offered; this was more evident in the antenatal booking card, as there were blank spaces under infant feeding counselling, meaning that they were not completed. Attitudes of nurses towards the implementation of the global steps for breastfeeding has been reported as one of the barriers for the successful implementation of MBFI, as nurses often feel that the strategy needs lot of change, and they are usually not prepared to change (Wieczorek et al. 2015:3). Some nurses, particularly old and experienced ones, demonstrated negative attitudes towards MBFI as they felt it was unnecessary new rubbish (Wieczorek et al. 2015:3). According to Leiter, Naegeli, and Walkley (2018:18), an institutional culture where breastfeeding is encouraged, accepted and widely practised would produce more positive breastfeeding attitudes and, in turn, could lead to an increase in breastfeeding initiation. The nurses’ attitudes towards breastfeeding has been reported as one of the determinants for breastfeeding, as nurses’ advice has great potential to either promote or discourage breastfeeding (Radzyminski & Callister 2015:102). There is little trace of studies on attitudes of nurses towards the implementation of MBFI in rural PHC facilities. Most studies from the available literature were conducted in hospital environments with physicians and patients, and most of these studies were in developed countries. Regardless of that, a paucity of literature focusing on the attitudes of nurses towards the implementation of MBFI in rural PHC facilities prevails in South Africa, and Makhuduthamga Municipality is not an exception. It is imperative to conduct such studies focusing on the attitudes of nurses, as they are the ones who provide first-line care to patients and ensure implementation of any proposed strategy by the NDoH in PHC facilities. Research that determines attitudes of nurses specific to MBFI implementation in rural facilities is imperative to enhance the development of support strategies that can be utilised by relevant stakeholders in the promotion of EBF and accreditation of all PHC facilities as mother-baby friendly facilities. Based on that, this study aimed to determine the attitudes of nurses towards the implementation of the MBFI strategy in the PHC facilities of Makhuduthamaga Municipality in Limpopo province.

## Aim of the study

The aim of the study was to determine the attitudes of nurses towards implementation of the MBFI in selected PHC facilities at Makhuduthamaga Municipality, Limpopo province.

## Significance of the study

This study may provide baseline data on the attitudes of nurses working in rural PHC facilities regarding the implementation of MBFI. Furthermore, these baseline results may assist in ascertaining whether the implementation of this strategy is feasible in other rural areas. The results of this study may help to inform the development of support and facilitative strategies that can be utilised by relevant stakeholders to promote positive attitudes towards the implementation of MBFI, which may increase breastfeeding rates and assist in accreditation of PHC facilities as mother-baby friendly institutions.

## Research methodology

### Study design

This study employed a quantitative descriptive design to determine nurses’ attitudes towards the implementation of MBFI in Makhuduthamaga Municipality. This approach enabled the researcher to analyse data using numerical information with statistical software to summarise and describe the results from the completed questionnaires. The descriptive design was utilised to gain more information about the attitudes of nurses towards the implementation of MBFI in Makhuduthamaga Municipality. Descriptive studies are conducted to provide an accurate portrayal of the sample characteristics or circumstances and frequency of a phenomenon’s occurrence (Burns, Grove & Gray 2015:212).

### Research setting

The study was conducted in 18 PHC facilities at Makhuduthamaga Municipality situated in the Greater Sekhukhune District, Limpopo province. The facilities were selected on the basis that they were being prepared for MBFI assessments for accreditation as mother-baby friendly facilities. In total, there are 21 PHC facilities in Makhuduthamaga Municipality: two are gateway clinics situated in two MBFI-accredited hospitals, one was accredited as a mother-baby friendly clinic in 2015 and the rest (18) of the clinics are non-accredited. There are approximately 176 nurses employed in the 18 non-accredited facilities. These include professional nurses (PNs = 91), enrolled nurses (ENs = 28) and enrolled nursing auxiliaries (ENAs = 57).

### Population and sampling

The population for this study consisted of nurses registered with the South African Nursing Council (SANC), working in the 18 non-accredited PHC facilities. A convenience sampling method was used in which nurses who volunteered to participate in the study were given questionnaires to complete. This is a non-probability sampling method that involves selection of the most readily available persons as participants in the study (Polit & Beck 2017:724).

### Data collection instrument, validity and reliability

The data collection instrument that was used in this study was a structured questionnaire developed by the researcher using the UNICEF/WHO 20-h breastfeeding manual, the UNICEF/WHO hospital self-appraisal tool, literature on attitudes of nurses towards BFHI/MBFI and researcher’s experience as MBFI assessor and trainer of trainees for the 40-h breastfeeding course. The questionnaire consisted of 27 items as follows: Section A had four items on the demographic characteristics such as age, gender, highest qualification and Section C had 19 four-point Likert scale questions on the attitudes of nurses towards MBFI. This section was subdivided into three subsections: four items on attitudes of nurses towards MBFI strategy implementation, nine on attitudes of nurses towards EBF and six on attitudes of nurses towards mother-friendly care. The questionnaire, written in English, took approximately 15–25 min to complete. The reason for using English was that all nurses had matric certificates and it was the only official language that nurses used for writing patient records.

The pretesting of the research instrument was done at Greater Tubatse Municipality with eight nurses. The pretest sample consisted of three PNs, three ENs and two ENAs. The results of the pretest study were discarded to avoid data contamination as it was undertaken to test the instrument for validity and reliability. The questionnaire was revised based on the pretest results, and the final questionnaire was checked by the study supervisors and the statistician before it was handed to respondents.

This was done to ensure face and content validity and also to ensure that it would yield the expected results. The experts also evaluated the readability and clarity of the items and instructions in the questionnaire during the process to address content validity (Polit & Beck 2017:310). The experts included MBFI assessors from the provincial health department and academics at the University of Venda.

### Data collection method

Data was collected using a self-administered structured questionnaire to gather information about the attitudes of nurses towards MBFI implementation in PHC facilities of Makhuduthamaga Municipality between April and May 2016. The self-administered questionnaire was accompanied by a leaflet that explained the aim of the study and the benefits of the study to all nurses. The researcher distributed the questionnaires and the information leaflets to all the selected PHC facilities, and the respondents were asked to drop the completed questionnaires in a box at the reception of each facility. The box was only opened and emptied by the researcher after 4 weeks of collection of the completed questionnaires.

### Data analysis

SPSS version 23 was used to analyse data with the assistance of a qualified statistician using a descriptive frequency analysis to describe the demographic data and attitudes of nurses towards implementation of MBFI. Data was presented through the use of tables derived from frequency analysis as indicated in the following section.

### Ethical considerations

Ethical clearance (SHS/15/PDC/34/0502) to conduct this study was granted by the University of Venda and permission was obtained from the Limpopo Department of Health. Informed written consent was acquired after explaining the research aims and benefits to the respondents, who then received information leaflets and signed consent forms. To ensure confidentiality, the completed questionnaires were placed in a box that would only be emptied by the researcher after 4 weeks. Furthermore, the researcher ensured anonymity by assigning a number to each questionnaire and respondents were instructed not to write their names on the questionnaire. Respondents completed the survey in their free time, at home or during lunch and were requested to return it within 4 weeks. The researcher kept the consent forms and questionnaires in a locked cupboard, and data information was only shared with the research supervisors and statistician. The researcher explained to the respondents that they were not being forced to participate in the study and that they were free to terminate if they felt uncomfortable, to ensure voluntary participation.

## Results

### Demographic characteristics

Nurses (*n* = 153) from 18 non-accredited PHC facilities participated in the study. The nurses who completed the questionnaire included 83 PNs, 25 ENs and 45 ENAs (Table 1 illustrates demographic data). The majority of nurses were between 31 and 40 years old. Most (*n* = 131, 86%) respondents in this study were females. The majority (*n* = 79, 62%) of nurses across the three nursing categories had diploma qualifications, followed by certificates for ENs and ENAs.

**TABLE 1:** Demographic characteristics of respondents (*n* = 153).

Variable	Professional nurses *N* = 83 *n* (%)	Enrolled nurses *N* = 25 *n* (%)	Enrolled nurse auxiliaries *N* = 45 *n* (%)
**Age**		
21–30 years	15 (18)	2 (8)	12 (27)
31–40 years	29 (35)	15 (60)	21 (47)
41–50 years	28 (34)	5 (20)	11 (24)
51 years and above	11 (13)	2 (12)	1 (2)
**Gender**			
Male	14 (17)	3 (12)	5 (11)
Female	69 (83)	22 (88)	40 (89)
**Academic qualification**			
Master’s degree	4 (5)	-	-
Bachelor’s degree	25 (30)	-	-
Diploma	45 (54)	17 (68)	17 (38)
Advanced diploma	9 (11)	-	-
Certificates	-	8 (32)	28 (62)

### Attitudes of nurses towards Mother-Baby Friendly Initiative strategy implementation

Four items determined the attitudes of nurses towards MBFI strategy implementation (see Table 2). Frequencies and percentages were calculated for each of the four items in this section. The majority of nurses, PNs (65, 78%), ENs (18, 72%) and ENAs (23, 51%) showed a positive attitude towards MBFI as they agreed that it increases breastfeeding rates. Most ENs (*n* = 20, 80%) and ENAs (39, 87%) had a negative attitude towards the MBFI strategy implementation because they indicated that it was time-consuming and different from their daily practice. Close to half of PNs (*n* = 36, 43%) also demonstrated negative attitudes as they concurred with the ENs and ENAs that MBFI was indeed time-consuming. The results revealed that 33 (40%) PNs, together with the majority of ENs (*n* = 18, 72%) and ENAs (*n* = 41, 91%), had a negative attitude towards MBFI strategy implementation as they reported that it was complicated and a serious burden to nurses. The majority of ENs (*n* = 15, 60%) and close to 100% of ENAs (*n* = 43, 96%) also demonstrated a negative attitude as they conceded that MBFI was for dieticians and midwives only. Most PNs (*n* = 53, 64%) showed a positive attitude towards MBFI strategy implementation because they disagreed that it was for dieticians and midwives only.

**TABLE 2:** Attitudes of nurses towards Mother-Baby Friendly Initiative strategy implementation (*n* = 153).

Statement	Professional nurses (*n* = 83)		Enrolled nurses (*n* = 25)		Enrolled nurse auxiliaries (*n* = 45)	
	Agree *n* (%)	Disagree *n* (%)	Agree *n* (%)	Disagree *n* (%)	Agree *n* (%)	Disagree *n* (%)
MBFI increases breastfeeding rates.	65 (78)	18 (22)	18 (72)	7 (28)	23 (51)	22 (49)
MBFI is time-consuming and different from our daily practice.	36 (43)	47 (57)	20 (80)	4 (20)	39 (87)	6 (13)
MBFI is complicated and a serious burden to nurses.	33 (40)	50 (60)	18 (72)	7 (28)	41 (91)	4 (9)
MBFI is for dieticians and midwives only.	30 (36)	53 (64)	15 (60)	10 (40)	43 (96)	2 (4)

MBFI, Mother-Baby Friendly Initiative.

### Attitudes of nurses towards exclusive breastfeeding

This section had nine items to determine the attitudes of nurses towards EBF (Table 3 illustrates the attitudes of nurses towards EBF). The majority of PNs (*n* = 70, 84%) and ENs (*n* = 21, 84%) demonstrated a positive attitude towards teaching pregnant women about benefits of breastfeeding, whereas 25 (56%) ENAs reported that they did not teach women about the benefits of EBF, which indicated a negative attitude. The majority of PNs (*n* = 67, 81%) and ENs (18, 72%) showed a positive attitude towards EBF because they agreed that babies less than 6 months old should be exclusively breastfed, while the majority (*n* = 30, 67%) of ENAs had a negative attitude because they disagreed. The results revealed that most PNs (*n* = 66, 80%) and ENs (*n* = 16, 64%) had a positive attitude towards the promotion, support and protection of breastfeeding, whereas on the other side a large group of ENAs (*n* = 34, 89%) had a negative attitude towards the promotion, support and protection of breastfeeding. Fifty-eight (70%) PNs, 15 (60%) ENs and only 7 (16%) ENAs were positive towards EBF because they indicated that it was the optimal feeding choice for any child. Regarding complementary feeding, 48 (58%) PNs and 17 (68%) ENs indicated that it should be done after 6 months, which also shows a positive attitude towards EBF and avoidance of mixed feeding, as any introduction of solid food or water before 6 months constitutes mixed feeding. Forty-six (55%) PNs, 11 (44%) ENs and only 5 (11%) ENAs demonstrated a negative attitude toward the use of dummies, as they reported that dummies undermined breastfeeding. Results show that most (*n* = 58, 70%) PNs disputed the statement that breastfeeding was for poor people, which shows a positive attitude towards breastfeeding as it is the best feeding option for all babies irrespective of the mother’s socio-economic status. Seventy-two percent (*n* = 18) of ENs and 78% (*n* = 35) of ENAs demonstrated a positive attitude towards use of mixed feeding practices because they agreed that babies aged less than 6 months should be given water, solid food and formula milk, while most PNs (*n* = 63, 76%) had a negative attitude towards mixed feeding, as they were against the introduction of water, solid food and formula milk before 6 months. With regard to continuation of breastfeeding among working mothers, most ENs (*n* = 19, 76%) and ENAs (*n* = 32, 71%) had negative attitudes towards the continuation of breastfeeding by working mothers as they agreed that working mothers were not allowed to breastfeed.

**TABLE 3:** Attitudes of nurses towards exclusive breastfeeding (*n* = 153).

Statement	Professional nurses (*n* = 83)		Enrolled nurses (*n* = 25)		Enrolled nurse auxiliaries (*n* = 45)	
	Agree *n* (%)	Disagree *n* (%)	Agree *n* (%)	Disagree *n* (%)	Agree *n* (%)	Disagree *n* (%)
I teach women about benefits of EBF.	70 (84)	13 (16)	21 (84)	4 (16)	20 (44)	25 (56)
Babies less than 6 months should be exclusively breastfed.	67 (81)	16 (19)	18 (72)	7 (28)	15 (33)	30 (67)
All nurses must promote, protect and support breastfeeding.	66 (80)	17 (20)	16 (64)	9 (36)	5 (11)	40 (89)
EBF is the optimal feeding choice for any child.	58 (70)	25 (30)	15 (60)	10 (40)	7 (16)	38 (84)
Complementary feeding should be done after 6 months only.	48 (58)	35 (42)	17 (68)	8 (32)	15 (33)	30 (67)
Dummies undermine breastfeeding.	46 (55)	37 (45)	11 (44)	14 (56)	5 (11)	40 (89)
Breastfeeding is for poor people.	25 (30)	58 (70)	14 (56)	11 (44)	30 (67)	15 (33)
Babies should be given water, solid food, formula milk before 6 months.	20 (24)	63 (76)	18 (72)	7 (28)	35 (78)	10 (22)
Working mothers are not allowed to breastfeed.	16 (19)	67 (81)	19 (76)	6 (24)	32 (71)	13 (29)

EBF, exclusive breastfeeding.

### Attitudes of nurses towards mother-friendly care

Six items were included in this section to determine the attitudes of nurses towards mother-friendly care (Table 4 illustrates the attitudes of nurses towards mother-friendly care). The majority of PNs (*n* = 62, 75%) and ENs (*n* = 13, 52%) had a positive attitude towards encouraging labouring women to perform minor exercise during labour because they agreed that a woman in labour should be encouraged to walk around during labour, while 28 (62%) ENAs had negative attitudes because they were against encouragement of labouring women to walk around during labour. Most PNs (*n* = 62, 75%) demonstrated a positive attitude towards companionship during labour; they agreed with the statement that a woman in labour must come with her companion for support. By contrast, the majority of ENs (*n* = 20, 80%) and ENAs (*n* = 37, 82%) disputed the statement, which shows a negative attitude. Further, 56% (*n* = 47) of PNs demonstrated a positive attitude towards allowing a labouring woman to assume the position of her choice, while 37 (44%) PNs, 19 (76%) ENs and 33 (73%) ENAs had a negative attitude towards allowing a woman in labour to assume the position of her choice. The majority of nurses (PNs = 53, 64%; ENs = 22, 88%; and ENAs = 43, 96%) had a negative attitude towards the avoidance of invasive procedures with labouring women. The results revealed that the majority of PNs (*n* = 65, 78%) and ENs (*n* = 14, 56%) had a negative attitude towards starving of women during labour. However, 22% (*n* = 18) of PNs, 11 (44%) ENs and 39 (87%) ENAs showed a positive attitude, as they agreed that a woman in labour should be starved. Regarding skin-to-skin contact between the mother and the baby, the majority (*n* = 63, 76%) PNs showed a positive attitude towards skin-to-skin contact, as they disagreed with the statement that it causes hypothermia. However, most ENs (*n* = 19, 76%) and ENAs (*n* = 37, 82%) had a negative attitude towards skin-to-skin contact between the mother and baby because they reported that it causes hypothermia.

**TABLE 4:** Attitudes of nurses towards mother-friendly care (*n* = 153).

Statement	Professional nurses (*n* = 83)		Enrolled nurses (*n* = 25)		Enrolled nurse auxiliaries (*n* = 45)	
	Agree *n* (%)	Disagree *n* (%)	Agree *n* (%)	Disagree *n* (%)	Agree *n* (%)	Disagree *n* (%)
A woman should be encouraged to walk around during labour.	62 (75)	21 (25)	13 (52)	12 (48)	17 (38)	28 (62)
A woman must come with her companion during labour for support.	62 (75)	21 (25)	5 (20)	20 (80)	8 (18)	37 (82)
A woman in labour should assume her position of choice.	47 (56)	37 (44)	6 (24)	19 (76)	12 (27)	33 (73)
I always avoid using invasive procedure like frequent PVs and early rupture of membranes in labour.	30 (36)	53 (64)	3 (12)	22 (88)	2 (4)	43 (96)
A woman in labour should be starved.	18 (22)	65 (78)	11 (44)	14 (56)	39 (87)	6 (13)
Skin-to-skin contact between the mother and baby causes hypothermia.	20 (24)	63 (76)	19 (76)	6 (24)	37 (82)	8 (18)

PVs, per vaginal examinations.

## Discussion

This study aimed to determine the attitudes of nurses towards the implementation of MBFI at Makhuduthamaga Municipality in Limpopo province. The majority of respondents in the sample were PNs (*n* = 83, 54%), followed by ENAs (*n* = 45, 30%), and ENs (*n* = 25, 16%) were the smallest group. This is because PHC facilities in South Africa are nurse-led and PNs perform most duties, from the dispensation of medication to the successful management of these facilities (Kredo et al. 2017:608). Enrolled nurses and ENAs are there to assist and support PNs in executing their duties. In this study, most nurses (PNs = 35%, ENs = 60%, ENAs = 47%) were aged between 31 and 40 years. The majority of respondents were females (87%) across the three nursing categories. Similarly, a study conducted in Vhembe on the knowledge and uptake of occupational post-exposure prophylaxis included more females than males (Makhado & Davhana-Maselesele 2016:4).

### Attitudes of nurses towards Mother-Baby Friendly Initiative strategy implementation

The majority of respondents in this study (PNs = 78%, ENs = 72%, ENAs = 51%) showed positive attitudes towards the MBFI strategy implementation, as they indicated that it increased breastfeeding rates. It is well documented within the available literature that adherence and implementation of the MBFI steps for successful breastfeeding has a positive impact on short-term, medium-term and longer-term breastfeeding outcomes across all topographies (Munn et al. 2016:685; Zakarija Grković et al. 2017:12249). Although the successful implementation of MBFI is associated with increased rates of breastfeeding, most ENs (80%), ENAs (87%) and more than a quarter of PNs (40%) in this study demonstrated negative attitudes towards MBFI, as they reported that it was time-consuming and different from their daily practice. This attitude could hinder the promotion of breastfeeding among mothers. The majority of two nursing categories, namely ENs (72%) and ENAs (91%), conveyed a negative attitude towards MBFI because they reported that it was complicated and a severe burden to nurses. The implementation of MBFI in PHC facilities does not necessarily provide an additional workload to nurses, but it is a better way of improving quality healthcare to mothers and babies. Promotion of breastfeeding by nurses falls within health promotion and prevention of diseases, so it is not a new thing or overwork for them. Nurses have four fundamental responsibilities, namely the promotion of health, the prevention of illness, the restoration of health and the alleviation of suffering (Harper & Maloney 2017:6; SANC 2013:5; White, Phakoe & Rispel 2015:26341).

Most nurses (57%) in this study showed a negative attitude towards MBFI, as they specified that it was for dieticians and midwives only. The MBFI strategy is not for dieticians only; it is a global initiative that aims to improve the quality of health outcomes for mothers and babies through successful breastfeeding (Pérez Escamilla, Martinez & Segura Pérez 2016:403). All nurses, irrespective of category, are required to abide by the principles of MBFI to improve breastfeeding rates and strive for the accreditation of their facilities as mother-baby friendly facilities. In a nutshell, most PNs had positive attitudes towards MBFI strategy implementation as compared to ENs and ENAs.

### Attitudes of nurses towards exclusive breastfeeding

Most respondents across the two nursing categories (PNs = 84%, ENs = 84%) indicated that they taught women about the benefits of breastfeeding, which showed that they had a positive attitude toward the promotion of breastfeeding, while a large group of ENAs (67%) demonstrated a negative attitude, as they did not teach women about the benefits of breastfeeding. This result is in line with WHO/UNICEF Step 3 of the 10 steps, which requires that all pregnant women be informed about the benefits and management of breastfeeding (UNICEF/WHO 2018:8). Similarly, Radzyminski and Callister (2015:107) in their study found that more than 75% of certified nurse-midwives in their study routinely provided information to women prenatally who enquired about infant feeding and always explained the benefits of breastfeeding. In this study, 100 (65%) nurses had a positive attitude towards EBF, as they agreed that babies less than 6 months should be exclusively breastfed, and 53 (35%) disagreed, which shows a negative attitude towards EBF. The WHO/UNICEF recommends that all babies aged less than 6 months be exclusively breastfed as breastfeeding has the capability to prevent childhood illnesses (UNICEF/WHO 2018:3; WHO 2013:5).

More than half of the respondents (*n* = 87, 57%) across the three nursing categories demonstrated a positive attitude towards breastfeeding as they indicated that all nurses must promote, protect and support breastfeeding, while most ENAs (*n* = 40, 89%) demonstrated negative attitude towards the statement. The National Infant and Young Child Feeding (IYCF) policy in South Africa explicitly states that all nurses and other healthcare professionals must promote, support and protect breastfeeding through continuous health education programmes to the communities (DoH 2013:16). Regarding complementary feeding, more than half of respondents (*n* = 80, 52%) across the three categories had a positive attitude towards complementary feeding, as they indicated that it should start after 6 months only. This is a positive response, which supports the avoidance of introduction of solid or additional foodstuffs before the age of 6 months, and this supports the notion that these nurses had more positive attitudes towards EBF. This is in line with the WHO recommendation that complementary feeding should only start at 6 months to meet the nutritional requirements of the baby (Aryeetey & Dyke 2018:2; Binns & Lee 2014:344). In this study, 62 (40%) respondents had a negative attitude toward the use of dummies, as they agreed that dummies undermine breastfeeding, whereas 91 (60%) demonstrated a positive attitude towards the use of dummies. This basically means the majority were in support of dummies, which are against the code of marketing for breast milk substitutes, which clearly states that dummies are not to be given to babies as sucking on the dummy or pacifier has no nutritive value and undermines breastfeeding (UNICEF/WHO 2018:21).

The majority of nurses in this study (*n* = 84, 55%) demonstrated a positive attitude towards promotion of breastfeeding across all economic classes because they disputed that breastfeeding was for poor people, meaning that these nurses would promote breastfeeding to every woman, whether rich or poor. What is of concern in this study is that 45% of respondents had a negative attitude towards this statement, as they agreed that it was for poor people, which then shows a negative attitude towards breastfeeding and is very undermining. Breastfeeding is an ideal feeding option for all babies irrespective of the mother’s socio-economic status. More than half (*n* = 80, 52%) had a positive attitude towards EBF because they were against giving water, solid food and formula milk to babies before 6 months. The correct practice is that babies younger than 6 months are not to be given any water, food or formula milk; the best feed for them is breast milk. The practice of giving babies water and solid food before the age of 6 months puts the baby at high risk for diarrheal infections and chest infections, and these often lead to death (Munn et al. 2016:222; Still, Marais & Hollis 2017:15). In this study, the majority of PNs (*n* = 67, 81%) disagreed with the view that working mothers are not allowed to breastfeed, which shows a positive attitude towards continued breastfeeding even if one is employed, whereas the majority of ENs (*n* = 19, 76%) and ENAs (*n* = 32, 71%) had a negative attitude towards continuation of breastfeeding among working mothers. Breastfeeding is regarded as the best feeding option for all babies irrespective of the mother’s socio-economic status, and all mothers are encouraged to breastfeed whether employed or unemployed (Mirkovic, Perrine & Scanlon 2016:236; Still et al. 2017:12336). There is no justification for not allowing working mothers to breastfeed. Because breastfeeding has been proven as the safest feeding method for all babies, it needs to be protected, promoted and supported even if the mother returns to work (Desmond & Meaney 2016:16; Mirkovic et al. 2016:233). A breastfeeding mother returning to work should receive support in making an individual plan, taking into account the age of her baby, childcare arrangements and the opportunities for the mother to express and store her milk (Haviland et al. 2015:120).

### Attitudes of nurses towards mother-friendly care

The addition of mother-friendly care to the 10 steps for successful breastfeeding was to ensure that all labouring women receive quality maternal care during labour and the delivery process. The focus of mother-friendly care is to give each labouring woman a positive birth experience and to avoid trauma, which impedes significantly the initiation and maintenance of breastfeeding. More than half (*n* = 92, 60%) of respondents showed a positive attitude towards encouraging a labouring woman to perform minor exercise, as they indicated that a woman in labour should be encouraged to walk around. This is in line with the components of mother-friendly care, which recommends that all labouring women without complications be encouraged to walk around during labour (Eckenrode 2018:41). The optimal way to give birth is through supporting the physiological process, as it brings about a positive birth experience, which tallies well with successful initiation of breastfeeding (Cheyney et al. 2014:107).

Regarding companionship in labour, the majority of respondents (*n* = 78, 51%) had negative attitudes towards birth companionship, as they disagreed with the statement that a ‘woman must come with her companion during labour’. This could mean that nurses won’t encourage male partners to accompany their female partners to the labour ward, which could mean loss of opportunity of male partners to receive breastfeeding counselling. The provision of support during the labour and delivery process helps with calming the mother (McGarry, Kroese & Cox 2016:26), facilitation of skin-to-skin contact and initiation of breastfeeding within 30 min after delivery of the baby (Schafer & Genna 2015:452). It is of concern that most nurses were against the promotion of companionship during labour despite the reported benefits such as the emotional and psychological support that it provides to the labouring woman. A study in Nigeria had similar results, where the majority of nurses (51.5%) declined the requests of women to have their male partners present because of the perception that they would disturb the birthing process (Adeniran et al. 2017:112). A study on perceptions about labour companionship at public teaching hospitals in the Arab states reported that women found comfort in the psychological support offered by a family member (Kabakian-Khasholian, El-Nemer & Bashour 2015:225). In contrast, the majority (*n* = 86, 56%) of nurses in this study demonstrated positive attitudes towards allowing a labouring woman to assume her position of choice, although 44% demonstrated negative attitudes towards allowing a woman to choose her position of choice. As part of mother-friendly care during birth, women are allowed to assume their positions of choice as they are in full control of their delivery process. This basically means nurses who had a positive attitude towards giving a woman freedom to assume her position would be helping in the reduction of maternal trauma and operative vaginal deliveries, which impact negatively on the initiation and duration of breastfeeding.

Regarding the avoidance of invasive procedures, only 35 (23%) respondents showed a positive attitude towards avoidance of invasive procedures during labour and the majority (*n* = 118, 77%) had a negative attitude towards avoidance of invasive procedures, as they disputed that they always avoided using invasive procedures like frequent per vaginal examinations (PVs) and early rupture of membranes in labour. This basically means these nurses were in support of invasive procedures during labour even though these are discouraged by the WHO/UNICEF (2018:12). Use of invasive procedures in labour would interfere with Step 4 of the revised WHO/UNICEF steps, which advocates for uninterrupted skin-to-skin contact and successful initiation of breastfeeding immediately after birth. Frequent PVs put the labouring woman at risk for chorioamnionitis and puerperal sepsis (Jansen et al. 2013:89), which impact negatively on breastfeeding initiation.

In this study, 85 (56%) respondents showed a negative attitude towards starving women in labour, and 68 (44%) demonstrated a positive attitude towards starvation during labour, as they agreed that a woman in labour should be starved. A more recent review found that women labouring in less-restrictive eating and drinking maternity wards had shorter labour and showed no difference in health outcomes (Ciardulli et al. 2017:476). Of concern is that 76 (50%) respondents had a negative attitude towards skin-to-skin contact between the mother and baby, as they indicated that it causes hypothermia. Skin-to-skin contact is the first step towards successful initiation of breastfeeding and its potential sensory stimulus includes newborn warming, tactile stimulation and promotion of breastfeeding (Hughes et al. 2015:486). Hypothermia can only occur if babies are not put skin-to-skin with their mothers, and if this happens those babies will die because of hypothermia. In order for babies to be kept warm and ensure that they can suck from their mothers’ breast, skin-to-skin contact should be implemented (Hughes et al. 2015:486).

## Recommendations

This study recommends that the Ministry of Health support PHC facilities and adopt strategies that will change the attitudes of nurses towards MBFI, such as the use of incentives to reward nurses who are adhering to MBFI principles. This study endorses the view that all nurses should teach the community about the benefits of breastfeeding. The PHC facilities should conduct in-service training sessions for all nurse categories to equip the ENs and ENAs. In-service training on mother-friendly care practices should be conducted to equip all nurses with skills and information to improve quality mother-friendly care, and each nurse should receive an orientation to the code of marketing for breast milk substitutes. Although ENs and ENAs do not conduct deliveries or perform midwifery procedures, basic counselling skills like encouraging a woman in labour to move or exercise and encouraging a woman to take light meals and fluids is a necessity. This information should be shared with them so that they may assist women correctly during labour. Nurse managers should ensure that all nurses working in the facilities are trained (20-h lactation course), as this training has been reported to have a great impact in changing nurses’ attitudes towards MBFI (Alakaam et al. 2018:323; Aryeetey & Dykes 2018:322; Balogun et al. 2017:540). The nurse managers should introduce a written IYCF policy and ensure that it is routinely communicated to all staff in their facility. Furthermore, they should ensure that their facilities implement the MBFI principles by encouraging all staff personnel to work towards attainment of the accreditation status as mother-baby friendly facility. This study recommends that similar studies be conducted in other parts of the country with nurses and non-health professionals. There is a need for qualitative studies to investigate the experiences and views of nurses about MBFI. In addition, studies are needed to evaluate PHC staff readiness for accreditation of their facilities for MBFI.

## Limitations of the study

The research was conducted in clinics in one rural municipality and the results cannot be generalised to clinics in urban municipalities.

## Conclusion

This study increased our insight on the attitudes of nurses towards the implementation of MBFI at PHC facilities in the Makhuduthamaga Municipality. The results revealed that most PNs had positive attitudes towards MBFI strategy implementation as compared to ENs and ENAs. This is a cause for concern as these two categories have almost the same number as PNs in the PHC facilities, and their negative attitude towards MBFI could hinder the promotion of breastfeeding, thereby preventing their clinics from becoming accredited facilities. The majority of PNs in this study disagreed with the statement that working mothers are not allowed to breastfeed, whereas the majority of ENs and ENAs agreed that working mothers are not allowed to breastfeed. The focus of MBFI strategy implementation is to strengthen and ensure that all mothers breastfeed their babies and that breastfeeding is promoted to all mothers irrespective of their socio-economic status. The majority of respondents across the three nursing categories had positive attitudes about EBF. With regard to MBFI strategy implementation and mother-friendly care, most PNs had high attitude scores as compared to ENs and ENAs. Therefore, it is important to devise strategies that can instil positive attitudes in all nursing categories towards MBFI strategy implementation if all the PHC facilities are to be accredited as mother-baby friendly facilities.
