# Corrigendum: Nurses’ attitudes towards the implementation of the Mother-Baby Friendly Initiative in selected primary healthcare facilities at Makhuduthamaga Municipality, Limpopo province

**DOI:** 10.4102/curationis.v42i1.2119

**Published:** 2019-12-06

**Authors:** Siyabulela E. Mgolozeli, Hilda N. Shilubane, Lunic B. Khoza

**Affiliations:** 1 Department of Advanced Nursing Science, University of Venda, Thohoyandou, South Africa

In the version of this article published earlier, in the reference list on page 9, the second author of the reference below was unintentionally omitted. The reference should read as follows:

Du Plessis, L.M. & Pereira, C.J., 2013, ‘Commitment and capacity for the support of breastfeeding in South Africa: A paediatric food-based dietary guideline’, *South African Journal of Clinical Nutrition* 26(3), S120–S128.

The sentence on page 2, first paragraph, is then updated to reflect the correct reference citation:

The renaming of BFHI to MBFI linked to Resolution 9 of the Tshwane Declaration (DoH 2014:33), which calls for community-based interventions to promote, protect and support breastfeeding (Du Plessis & Pereira 2013:125).

This correction does not alter the study’s findings of significance or overall interpretation of the study results. The authors apologise for any inconvenience caused.

